# A predictive model for upper gastrointestinal bleeding in patients with acute myocardial infarction complicated by cardiogenic shock during hospitalization

**DOI:** 10.3389/fcvm.2025.1662067

**Published:** 2025-10-02

**Authors:** Fei Dong, Ying Zhou, Yufei Zhao, Yunqiang Zhang, Haiqing Liang, Yu Song, Rui Jing

**Affiliations:** ^1^ Department of Heart Failure, TEDA International Cardiovascular Hospital, Tianjin University, Tianjin, China; ^2^ Cardiovascular Intensive Care Unit, TEDA International Cardiovascular Hospital, Tianjin University, Tianjin, China

**Keywords:** acute myocardial infarction, cardiogenic shock, upper gastrointestinal bleeding, predictive model, prognosis

## Abstract

**Objective:**

To explore the current status and characteristics of upper gastrointestinal bleeding (UGIB) in patients with acute myocardial infarction complicated by cardiogenic shock (AMICS) following emergency percutaneous coronary intervention (PCI), and to develop and validate a predictive model based on baseline risk factors at the time of admission.

**Methods:**

We selected patients diagnosed with AMICS who underwent emergency PCI. Patients were categorized into the non-bleeding group and the bleeding group based on the occurrence of UGIB during hospitalization. Logistic regression analysis was employed to construct a predictive model for UGIB based on baseline risk factors at admission.

**Results:**

A total of 253 patients were included in the study, of whom 58 experienced UGIB, resulting in an incidence rate of 22.9%. Univariate analysis indicated that the levels of uric acid, lactate, and alanine aminotransferase (ALT) were higher in the bleeding group compared to the non-bleeding group. Conversely, the estimated glomerular filtration rate (eGFR), left ventricular ejection fraction (LVEF), and albumin were lower in the bleeding group. Additionally, the bleeding group had a higher stage of American Society for Cardiovascular Angiography and Interventions-Cardiogenic Shock(SCAI-CS). The least absolute shrinkage and selection operator (LASSO) regression identified 5 non-zero coefficient variables, and the variance inflation factor (VIF) test excluded the collinearity relationships among these 5 variables. Continuous variables were converted into categorical variables and assigned as follows: albumin was categorized based on whether below 35 g/L, indicating hypoproteinemia; eGFR was categorized based on whether below 45 ml/(min·1.73 m^2^), indicating moderate to severe renal function decline;The receiver operating characteristic curve (ROC) curve was employed to identify the node with the highest Youden index, which determines the cut-off value: For lactate, the corresponding value is 6.95 mmol/L, rounded to 7 mmol/L. For LVEF, the corresponding value is 35.5%, rounded to 36%.Multivariate logistic regression analysis identified 3 risk factors for UGIB following AMICS emergency PCI: baseline SCAI-CS stage D + E, baseline eGFR < 45 ml/(min·1.73 m^2^), and baseline LVEF < 36% (*P* < 0.05). The predictive model based on multivariate logistic regression results, demonstrated good fit according to the Hosmer-Lemeshow test (*χ*² = 6.968, *P* = 0.324). The model achieved an AUC of 0.768 [95% CI (0.700, 0.837)] in predicting UGIB in patients undergoing emergency PCI for AMICS, with a sensitivity of 87.9% [95% CI (0.700, 0.837)] and a specificity of 52.3% [95% CI (27.8%, 61.2%)]. The DeLong test indicated that the AUC of the predictive model was superior to that of any individual indicator (*P* < 0.05), and the DCA curve confirmed the model's clinical utility. Recent Prognosis: The mortality rate during hospitalization in the bleeding group was significantly higher than that in the non-bleeding group (63.8% vs. 29.7%, *P* < 0.05). Additionally, the duration of hospital stays for surviving patients in the bleeding group was significantly longer than that in the non-bleeding group [19 (14,37) vs. 12 (9,16), (*P* < 0.001)].

**Conclusion:**

High baseline SCAI-CS stage, poor kidney function, and low LVEF are independent risk factors for UGIB during hospitalization. The constructed predictive model demonstrates high predictive efficacy.

## 1 Introduction

Cardiogenic shock (CS) is a clinical syndrome characterized by inadequate perfusion of terminal organs due to cardiac pump failure. It represents the most severe form of acute heart failure. Approximately 10% of patients with acute ST-segment elevation myocardial infarction (STEMI) develop CS, with an associated early mortality rate of 40%–50% (1, 2). In patients with acute coronary syndrome (ACS) complicated by CS, emergency percutaneous coronary intervention (PCI) or coronary artery bypass grafting (CABG) can improve survival, regardless of symptom onset timing (3–6). Although intra-aortic balloon pump (IABP) and extracorporeal membrane oxygenation (ECMO) have not demonstrated survival benefits in patients with acute myocardial infarction complicated by cardiogenic shock (AMICS), they remain valuable for hemodynamic stabilization and facilitating PCI. In particular, for patients with ACS complicated by mechanical complications, percutaneous mechanical circulatory support (pMCS) can serve as a bridge to recovery or provide durable support (7–14). Our hospital is equipped with a excellent Chest Pain Center and a specialized critical heart team, enabling us to perform emergency coronary revascularization and to proficiently utilize pMCS for AMICS patients in accordance with current guidelines.

Although patients with AMICS undergo early revascularization, the early mortality rate remains high. According to reference (15), 54% of these patients die from cardiogenic causes, 24% from brain injury, 20% from multi-organ dysfunction, and an additional 2% from other causes such as sepsis, respiratory failure, and bleeding.

Therefore, patients with AMICS urgently require management of various complications while maintaining hemodynamic stability. Bleeding is a common complication in AMICS, which can be classified based on the bleeding site into puncture site bleeding and non-puncture site bleeding. Puncture site bleeding is frequently associated with local vascular conditions, puncture techniques, and the use of pMCS.

Upper gastrointestinal bleeding (UGIB) following emergency PCI for AMICS complicates treatment. Significant bleeding can exacerbate hemodynamic instability and necessitate reduction or cessation of antithrombotic therapy. Bleeding-induced activation of the coagulation system and transfusions may lead to hypercoagulability, increasing the risk of reinfarction and embolism. Currently, there are no standardized guidelines for managing UGIB in the context of acute myocardial infarction (AMI). Clinical strategies primarily involve balancing antithrombotic and hemostatic interventions based on relevant scoring systems, such as the DAPT score and BARC bleeding classification. However, these scores are mainly designed for long-term management and are less applicable during the acute phase of AMICS, with limited research addressing UGIB in this setting.

This study aims to analyze the incidence, clinical characteristics, prognosis, and risk factors of UGIB in patients after AMICS undergoing emergency PCI during hospitalization. Additionally, a predictive model will be developed and validated based on baseline admission risk factors. The findings are expected to enhance understanding of this condition, facilitate early identification of high-risk patients, enable timely preventive measures, and ultimately reduce bleeding-related adverse outcomes.

## 2 Methods

### 2.1 Study design and study subjects

This study is a single-center, retrospective cohort analysis involving 253 patients treated at TEDA International Cardiovascular Hospital, all of whom were diagnosed with AMICS and underwent emergency PCI between January 2018 and December 2024. Patients were categorized into two groups based on the occurrence of UGIB during hospitalization: the non-bleeding group (*n* = 195) and the bleeding group (*n* = 58).

**Inclusion Criteria:**

1. Patients diagnosed with AMICS who underwent emergency PCI at the TEDA International Cardiovascular Hospital. The emergency PCI procedures included percutaneous coronary artery endovascular surgery, coronary stent implantation, and coronary thrombosis aspiration.
2. Diagnostic criteria for CS: Systolic blood pressure (SBP) ≤90 mmHg (1 mmHg = 0.133 kPa) or a mean arterial pressure (MAP) drop ≥30 mmHg; a sustained SBP decrease of ≥60 mmHg for at least 30 min.Cardiac index (CI) ≤2.2 L/(min·m^2^); pulmonary capillary wedge pressure ≥ 15 mmHg; Signs of organ hypoperfusion include cyanosis, limb chills, and decreased urine output < [0.5 ml/(kg·h)]. CS stage was based on the standard of the American Society for Cardiovascular Angiography and Interventions (SCAI) (16):
  - Stage A (At Risk): Patients without symptoms or signs of CS but at risk.
  - Stage B (Beginning): Patients with relatively low blood pressure and no signs of inadequate perfusion.
  - Stage C (Classic): Patients exhibiting low perfusion requiring initial intervention (inotropes, pharmacologic support, or mechanical circulatory support) after volume resuscitation to restore perfusion.
  - Stage D (Deteriorating): Patients whose initial stabilization is unstable, necessitating further escalation of treatment.
  - Stage E (Extremis): Patients with refractory circulatory failure, often in cardiac arrest or requiring multiple concurrent interventions, including ECMO-assisted cardiopulmonary resuscitation.
3. UGIB: Based on the “Expert Suggestions for the Prevention and Treatment of Stress Ulcers” (2018 Edition) (17) and the “Expert Consensus for the Emergency Diagnosis and Treatment of Acute Upper Gastrointestinal Hemorrhage” (2020 Edition) (18), UGIB is defined as follows:
  - Clinical manifestations include vomiting, melena, tarry stool, or gastric tube drainage of brown or bloody gastric contents, with or without symptoms such as dizziness, palpitations, oliguria, pallor of skin and mucous membranes, tachycardia, hypotension, and altered consciousness. Additionally, gastric juice, vomit, or fecal samples may test positive for occult blood.
  - The above symptoms are absent, except for positive stool occult blood or gastric juice tests. After multidisciplinary evaluation, bleeding from the oral cavity, nasopharynx, or other sites is excluded. In cases where bleeding is due to hemorrhoids, mucus, blood-stained stool, pus, gastroenteritis, or other causes, a decrease in hemoglobin concentration of ≥20 g/L is observed for unknown reasons.
4. Grading the Severity of UGIB according to the GUSTO Bleeding Classification Criteria (19):
  - Severe bleeding: Life-threatening or severe bleeding.
  - Moderate bleeding: Bleeding requiring blood transfusion but not causing hemodynamic instability.
  - Mild bleeding: Bleeding that does not meet the criteria for severe or moderate bleeding.

**Exclusion criteria:**

1. Patients with esophageal or gastric varices;
2. Patients with a tendency to bleed due to coagulation dysfunction, platelet abnormalities, or abnormal platelet counts upon admission;
3. Patients exhibiting symptoms of gastrointestinal bleeding, such as hematemesis or melena, or with a recent history of untreated gastrointestinal bleeding diseases.

This study was approved by our ethics committee.

### 2.2 Study protocol

1. Patients diagnosed with AMICS will receive liquid resuscitation, vasoactive agents, mechanical ventilation, pMCS, and other supportive therapies tailored to their clinical condition to maintain vital signs.
2. Preoperative oral loading of dual antiplatelet agents—aspirin 300 mg and clopidogrel 300 mg or ticagrelor 180 mg—is recommended. Postoperative antiplatelet therapy includes aspirin 100 mg once daily and either clopidogrel 75 mg once daily or ticagrelor 90 mg twice daily. The antiplatelet regimen should be adjusted according to the patient's bleeding risk. Routine administration of statins is advised for lipid regulation, along with other secondary prevention strategies for coronary heart disease.
3. Coronary angiography and PCI are performed using standard techniques. The PCI strategy is determined by the operator based on the patient's condition and is followed by transfer to the cardiac intensive care unit post-procedure.
4. Proton pump inhibitor(PPI) prophylaxis was administered post-PCI upon transfer to the CCU, with no preoperative PPI use; this timing was determined by the hospital's emergency PCI pathway, which prioritizes rapid revascularization over prophylactic medications. Routine PPI prophylaxis: Following PCI, all patients were transferred to the CCU and subsequently administered oral Pantoprazole 40 mg once daily. PPI adjustment for UGIB: In cases where patients developed UGIB, the oral PPI was promptly switched to intravenous Pantoprazole at a dosage of 80 mg daily.
5. Patients with UGIB should undergo an assessment of bleeding severity. In cases of moderate or severe bleeding, antiplatelet drugs should be promptly discontinued. For mild bleeding, therapy may be temporarily halted or limited to a single antiplatelet agent, with adjustments made based on clinical changes. All patients with UGIB should receive symptomatic treatment, which includes fasting, gastrointestinal decompression, and the use of PPIs to inhibit gastric acid secretion and protect the gastric mucosa. Hemostatic agents may be administered orally or *via* a gastric tube, depending on the patient's condition.

### 2.3 Observation indicators

1. Collect baseline data for both groups of patients upon hospital admission. This data should include gender, age, body mass index (BMI), medical history, personal history, estimated glomerular filtration rate (eGFR), total leukocyte count, hemoglobin levels, platelet count, C-reactive protein, alanine aminotransferase (ALT), baseline systolic blood pressure, baseline heart rate, D-dimer levels, lactic acid, uric acid, total cholesterol, triglycerides, low-density lipoprotein cholesterol, left ventricular ejection fraction (LVEF), plasma B-type natriuretic peptide (BNP), results from coronary angiography, and targeted blood vessels for PCI.
2. Prognosis: Document the in-hospital mortality rates and the duration of hospitalization for patients who survive in both groups.

### 2.4 Data cleaning

The data contains fewer than 5% missing values, which will be addressed through imputation. For continuous variables exhibiting a normal distribution, the mean will be used for imputation; otherwise, the median will be applied. For categorical variables, the most frequent value will be utilized for imputation.

### 2.5 Statistical methods

**Figure 1:**
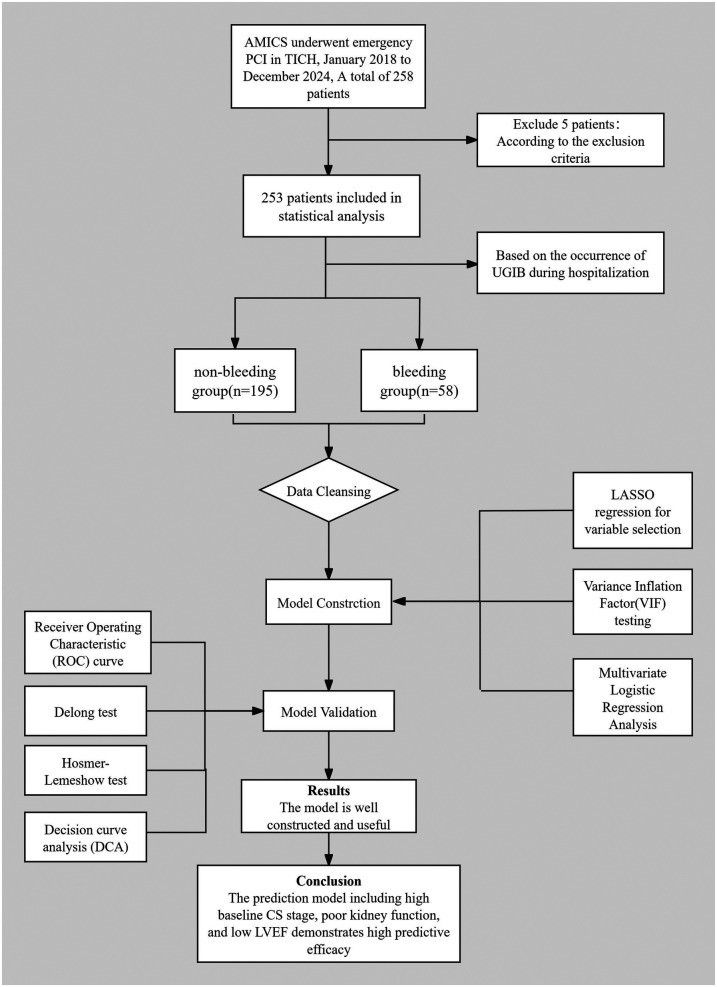
Prognostic flow diagram.

Data analysis was conducted using SPSS version 27.0 and R version 4.5.1. Continuous variables that followed a normal distribution were expressed as mean ± standard deviation (SD), and comparisons between groups were performed using the *t*-test. Variables that did not follow a normal distribution were presented as medians (P_25_, P_75_), and the Mann–Whitney *U* test was utilized for inter-group comparisons. Categorical data were described using frequencies (*n*) and percentages (%), and comparisons were made using the chi-square test or Fisher's exact test.Variables that demonstrated statistically significant differences in univariate analysis were subjected to least absolute shrinkage and selection operator(LASSO) regression to identify key predictors. These selected variables were then assessed using the variance inflation factor (VIF) test to address multicollinearity issues. Finally, the refined set of variables underwent multivariate logistic regression analysis. The model's predictive efficacy and fit were evaluated using the receiver operating characteristic (ROC) curve and the Hosmer-Lemeshow goodness-of-fit test. The area under the curve (AUC) was compared using the DeLong test. Decision curve analysis (DCA) was performed to assess the clinical utility of the model. The length of hospital stay was compared between the two groups. *P* < 0.05 was considered statistically significant. All tests were two-sided, with a significance level set at *α* = 0.05. A prognostic flow diagram is presented in Figure 1.

## 3 Results

### 3.1 Univariate analysis

In this study, 253 AMICS patients undergoing emergency PCI were included. Among these, 58 patients experienced UGIB during hospitalization, resulting in an incidence rate of 22.9%. Of these, 33 cases (13.0%) were classified as mild bleeding, 22 cases (8.7%) as moderate bleeding requiring transfusion, and 3 cases (1.2%) as severe bleeding, which was life-threatening. All three patients with severe bleeding exhibited abnormal coagulation function and multiple bleeding sites.

The univariate analysis indicated that the levels of uric acid, lactate, and ALT were higher in the bleeding group compared to the non-bleeding group. Conversely, the levels of eGFR, LVEF, and albumin were lower in the bleeding group. Additionally, the bleeding group exhibited a higher stage of SCAI-CS. All differences were statistically significant (*P* < 0.05) (Table 1).

**Table 1:** Univariate analysis of UGIB in AMICS patients after emergency PCI during hospitalization.

Items	Non-bleeding group	Bleeding group	*t*/*χ*^2^/*Z value*	*P*
	(*n* = 195)	(*n* = 58)		
Age (years)	67 (57,77)	68 (56,77)	−0.159	0.873
Male [Case (%)]	134 (68.7)	43 (74.1)	0.625	0.429
BMI (kg/m^2^)	24.61 (22.57,26.56)	25.39 (22.64,27.3)	−0.941	0.347
Past medical history and personal history				
Malignant tumor [case (%)]	7 (3.6)	0 (0)	^⋆^	0.357
History of stroke [case (%)]	28 (14.4)	9 (15.5)	0.048	0.827
Hypertension [case (%)]	99 (50.8)	30 (51.7)	0.016	0.898
Diabetes [case (%)]	59 (30.3)	20 (34.5)	0.372	0.542
Smoking history [cases (%)]	103 (52.8)	26 (44.8)	1.143	0.285
History of Peptic ulcer [case (%)]	6 (3.1)	2 (3.5)	0.000	1.000
Baseline SBP (mmHg)	80 (70,90)	78 (60.75,89.25)	−1.862	0.063
Baseline heart rate (beats/min)	81 (50,108)	84.5 (50,104)	−0.077	0.939
D-dimer (ng/ml)			2.210	0.530
<500 [case(%)]	85 (43.6)	25 (43.1)		
500–1,000 [case(%)]	42 (21.5)	8 (13.8)		
1,000–5,000 [case(%)]	58 (29.7)	21 (36.2)		
>5,000 [case(%)]	10 (5.1)	4 (6.9)		
eGFR [ml/(min·1.73 m^2^)]	71.77 (54.18,91.77)	59.28 (39.9,75.71)	−3.400	<0.001
Uric Acid (mmol/L)	395 (323,464)	464 (300,535)	−3.710	<0.001
WBC (*10^9^/L)	11.2 (9,15.2)	12.1 (10.3,15.3)	−1.616	0.106
HGB (g/L)	137.7 ± 21.6	136.8 ± 24.0	0.27	0.787
PLT (×10^9^/L)	238 (197,282)	230 (171,276)	−1.478	0.139
CRP (mg/L)	7.8 (2.1,31.4)	12.6 (2.8,48.8)	−1.295	0.195
Lactate (mmol/L)	4.6 (2.8,7.9)	7.3 (3.9,15.3)	−3.724	<0.001
Plasma BNP (pg/ml)	142 (34,559)	204 (47,643)	−0.950	0.342
ALT (U/L)	90 (42,169)	157 (90,256)	−4.249	<0.001
Total cholesterol (mmol/L)	4.2 (3.6,4.8)	4.1 (3.1,5.1)	−0.970	0.332
Triglyceride (mmol/L)	1.03 (0.72,1.49)	1.12 (0.67,1.45)	−0.047	0.962
LDL-C (mmol/L)	2.60 (2.06,3.21)	2.60 (1.88,3.53)	−0.0.128	0.898
Albumin (g/L)	37 (35,40)	36 (33,39)	−2.773	0.006
LVEF (%)	42.4 ± 11.1	36.3 ± 13.7	3.461	<0.001
LM lesion [case (%)]	22 (11.3)	12 (20.7)	3.401	0.065
History of cardiac arrest [case (%)]	69 (35.4)	23 (39.7)	0.352	0.553
Anticoagulation [case (%)]	32 (16.4)	10 (17.2)	0.022	0.881
P2Y₁₂ receptor antagonist			0.026	0.871
Ticagrelor [case (%)]	20 (34.5)	65 (33.3)		
SCAI-CS stage			21.830	<0.001
Stage C [case (%)]	127 (65.1)	18 (31.0)		
Stage D [case (%)]	50 (25.6)	27 (46.6)		
Stage E [case (%)]	18 (9.2)	13 (22.4)		

Note: ^⋆^Fisher's exact test was used.

The comparison across the three SCAI-CS stages was performed using the Bonferroni correction (*α* = 0.05/3 ≈ 0.0167). The results indicated significant differences between stage C and stage D (*χ*² = 15.966, *P* < 0.001), and between stage C and stage E (*χ*² = 15.339, *P* < 0.001). No significant difference was observed between stages D and E (*χ*² = 0.447, *P* = 0.504). Consequently, stages D and E were combined into stage D + E for subsequent analysis.

A significant difference was found between stage C and the stage D + E (*χ*² = 21.239, *P* < 0.001).

### 3.2 Variable selection

**Figure 2:**
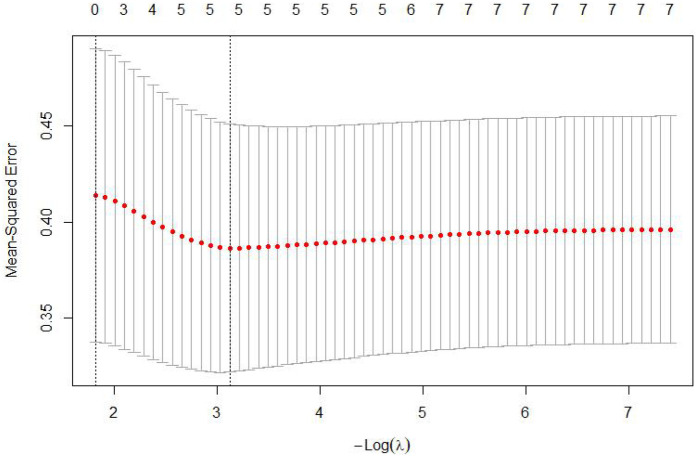
Cross-validation curve of LASSO regression.

**Figure 3:**
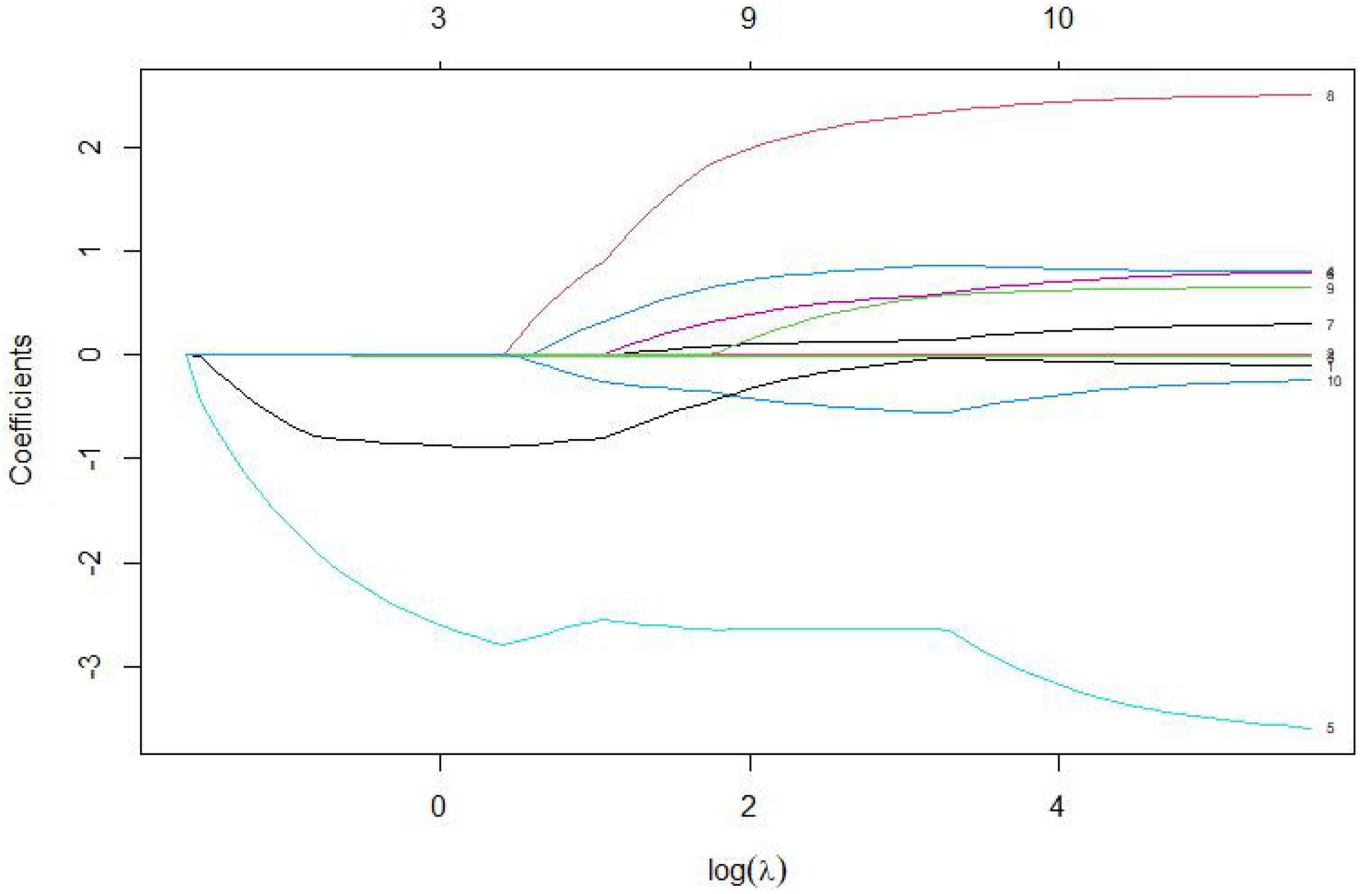
Coefficient path diagram of risk variables.

**Figure 4:**
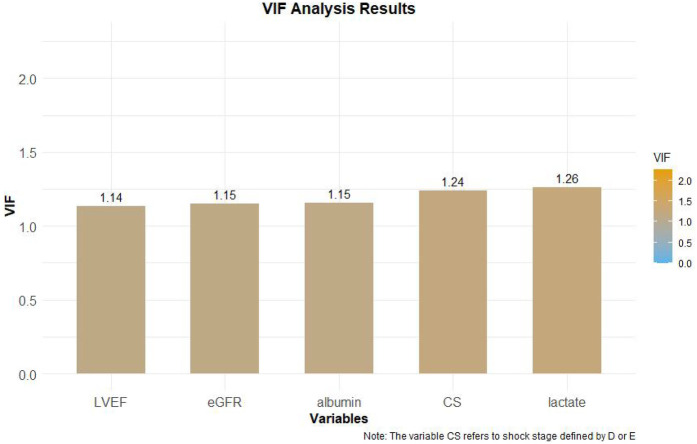
VIF test results.

The 7 variables identified as statistically significant through univariate analysis were subjected to LASSO regression for dimensionality reduction and initial variable screening. A 10-fold cross-validation was employed to determine the optimal *λ* value, which minimized the cross-validation error. Variables with non-zero regression coefficients corresponding to the optimal *λ* value were further analyzed statistically. The LASSO regression results indicated a minimum error value of 0.044. At this point, the variables with non-zero regression coefficients were lactate, eGFR, LVEF, SCAI-CS stage, and albumin (Figures 2, 3). A variance inflation factor (VIF) test was conducted on the aforementioned 5 variables, and the results indicated no multicollinearity issues among them (Figure 4).

### 3.3 Variable assignment

Continuous variables were converted into categorical variables and assigned as follows: albumin was categorized based on whether below 35 g/L, indicating hypoproteinemia; eGFR was categorized based on whether below 45 ml/(min·1.73 m^2^), indicating moderate to severe renal function decline;The ROC curve was employed to identify the node with the highest Youden index, which determines the cut-off value: For lactate, the corresponding value is 6.95 mmol/L, rounded to 7 mmol/L. For LVEF, the corresponding value is 35.5%, rounded to 36%.

The specific assignments are as follows: SCAI-CS stage (C = 0, D + E = 1), lactate (<7 mmol/L = 0, ≥7 mmol/L = 1), LVEF (≥ 36% = 0, <36% = 1), hypoproteinemia (≥35 g/L = 0, <35 g/L = 1), and eGFR [≥45 ml/(min·1.73 m^2^) = 0, <45 ml/(min·1.73 m^2^) = 1].

### 3.4 Multivariate logistic analysis, construction and validation of a predictive model for UGIB in patients undergoing emergency PCI for AMICS

After conducting the univariate analysis and selecting variables, those with statistical significance were transformed into binary categorical variables and utilized as independent variables. The occurrence of UGIB after emergency PCI for AMICS was set as the dependent variable (no bleeding = 0, bleeding = 1). A binary multivariate logistic regression analysis was then conducted. The results identified the following as independent risk factors (*P* < 0.05): baseline SCAI-CS stage D + E, baseline eGFR < 45 ml/(min·1.73 m^2^), and baseline LVEF < 36% (Table 2).

**Table 2:** Multivariate logistic analysis of UGIB in AMICS patients after emergency PCI during hospitalization.

Risk factors	*Β* value	*Waldχ*^2^ value	*OR* value 95% CI	*P* value
SCAI-CS D + E	1.023	8.028	2.781 (1.371,5.642)	0.005
eGFR < 45 ml/(min·1.73 m^2^)	0.991	6.322	2.695 (1.244,5.836)	0.012
LVEF < 36%	0.908	7.200	2.479 (1.227,4.810)	0.007

**Figure 5:**
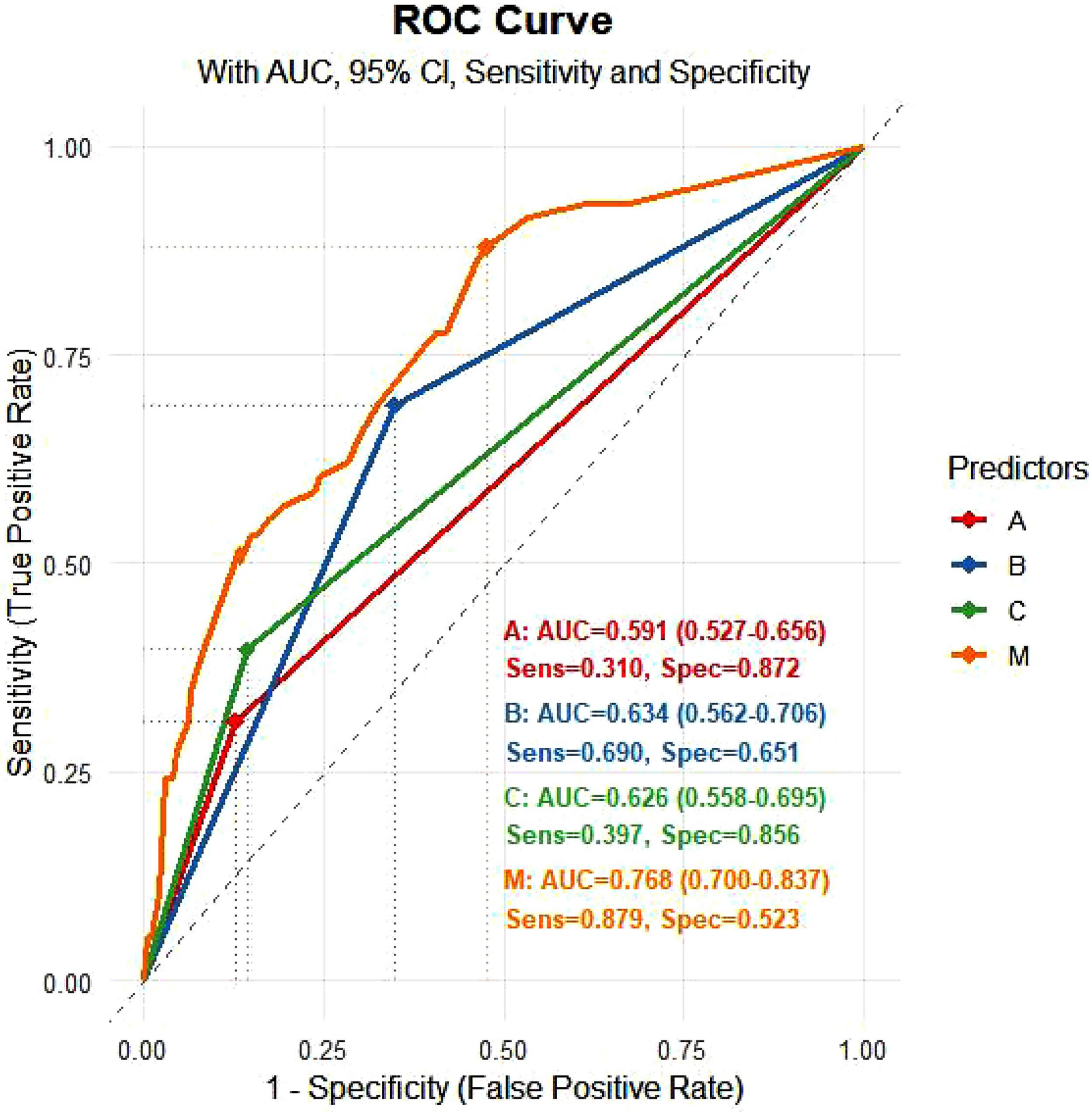
ROC curve for logistic regression (validation). **(A)**: eGFR < 45 ml/(min·1.73 m²); **(B)**: LVEF < 36%; **(C)**: SCAI-CS D + E; M: prediction probability.

The Hosmer-Lemeshow test yielded a *χ*² value of 6.968 with a *P* value of 0.324, indicating a good fit for the model. The ROC for predicting in-hospital UGIB indicated that for patients with baseline SCAI-CS stage D + E, baseline eGFR < 45 ml/(min·1.73 m^2^), baseline LVEF < 36%, and the combined indicator were 0.670 [95% CI (0.591, 0.750)], 0.654 [95% CI (0.503, 0.679)], 0.634 [95% CI (0.550, 0.718)], and 0.768 [95% CI (0.700, 0.837)], respectively (Figure 5). At the optimal cut-off for the combined indicator, the sensitivity was 87.9% [95% CI (0.700, 0.837)] and the specificity was 52.3% [95% CI (27.8%, 61.2%)].

The Delong test demonstrated that the AUC of the predictive model was significantly higher than that of any individual indicator (all *P* < 0.05), indicating superior diagnostic efficacy.

**Figure 6:**
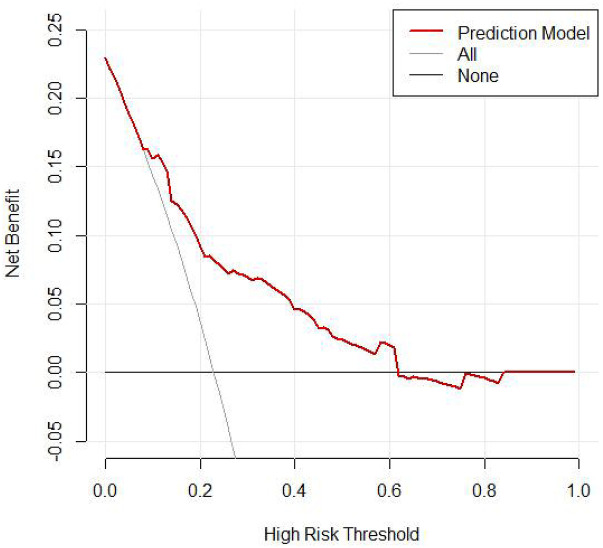
DCA curve for logistic regression (validation).

According to the DCA, this combined prediction model offers greater clinical utility when the risk threshold ranges from 0%–61% (Figure 6).

### 3.5 Short-term prognosis

The results indicated that the in-hospital mortality rate for patients in the bleeding group was significantly higher than that of the non-bleeding group (29.7% vs. 63.8%), with a statistically significant difference (*P* < 0.05). Additionally, the duration of hospital stay for surviving patients in the bleeding group was significantly longer than that of the non-bleeding group (Table 3), and this difference was also statistically significant (*P* < 0.05).

**Table 3:** Comparison of length of hospital stay among patients who survived the hospital.

Items	Non-bleeding group	Bleeding group	*Z* value	*P* value
	(*n* = 137)	(*n* = 21)		
Hospital stays	12 (9,16)	19 (14,37)	−3.871	<0.001

## 4 Discussion

### 4.1 The incidence of UGIB during hospitalization for AMICS after emergency PCI

Liang Zhong et al. (20) found that among the 5,868 AMI patients included in their study, gastrointestinal bleeding occurred during hospitalization. The study identified a history of diabetes, Killip class IV, and renal insufficiency as independent risk factors for gastrointestinal bleeding. A meta-analysis of risk factors for gastrointestinal bleeding in patients following PCI (21) included 16 studies involving 584,903 patients. The results indicated that age ≥60 years, a history of smoking, hypertension, renal insufficiency, a previous history of digestive tract disease, combined with CS, a diagnosis of ACS, a decline in platelet count, a decrease in baseline hemoglobin, and the use of glycoprotein IIb/IIIa inhibitors are significant risk factors for gastrointestinal bleeding in patients after PCI. Conversely, the use of PPIs was identified as a protective factor. Tian Xue et al. (22) analyzed 3,207 AMI patients at Anzhen Hospital, reporting an incidence of gastrointestinal bleeding during hospitalization of 6.4%. Their study identified combined with CS, a history of gastrointestinal bleeding, and elevated creatinine levels as risk factors for gastrointestinal bleeding in AMI patients.

Current research primarily focuses on the study of ACS and its association with gastrointestinal bleeding. These studies indicate that conditions such as hypotension and shock are significant risk factors for bleeding and are also correlated with patient prognosis. However, there is a scarcity of research on in-hospital UGIB in patients with AMICS following emergency PCI. This study specifically targets critically ill ACS patients and seeks to investigate the incidence, severity, and risk factors of UGIB in AMICS patients post-emergency PCI. It also aims to analyze the potential impact of UGIB on the prognosis of these patients. By focusing on this high-risk population, our research addresses a notable gap in existing studies regarding gastrointestinal bleeding in AMI patients. Our research shows that the incidence of UGIB during hospitalization for AMICS patients after emergency PCI was 22.9%, which is significantly higher than the reported incidence among AMI patients.

AMICS leads to a decrease in cardiac output, activation of the sympathetic nervous system, and stimulation of the renin-angiotensin-aldosterone system. These responses promote vascular constriction and cause intense contraction of gastrointestinal blood vessels, prioritizing perfusion to vital organs such as the heart and brain. The gastrointestinal mucosa is highly sensitive to ischemia and hypoxia; prolonged ischemia damages mucosal cells, impairs their function, and weakens the mucosal barrier. This enhances the susceptibility of the gastric mucosa to the corrosive effects of gastric acid and pepsin, leading to erosion, ulceration, and potentially bleeding in severe cases (23, 24). Additionally, reperfusion after PCI may exacerbate systemic oxidative stress, further damaging the gastrointestinal mucosa (25).

### 4.2 The predictive model and risk factors of UGIB for AMICS after emergency PCI

Our predictive model constructed for in-hospital UGIB for AMICS after emergency PCI identifies the following independent risk factors: baseline SCAI-CS stage D + E, baseline eGFR < 45 ml/(min·1.73 m^2^), and baseline LVEF < 36%.

Severe shock activates systemic inflammatory responses, increases gastric acid secretion, reduces mucosal defenses, and predisposes to stress ulcer formation (26). Patients with CS stage D or E, according to the SCAI classification, experience more severe systemic hypoperfusion and are more likely to receive higher doses of antithrombotic agents or pMCS. These stages are often associated with disseminated intravascular coagulation or platelet depletion, which elevate the bleeding risk (27).

An eGFR below 45 ml/(min·1.73 m^2^) signifies moderate to severe renal insufficiency, resulting in the accumulation of uremic toxins, impaired platelet aggregation, and adhesion functions, thereby increasing the risk of bleeding (28). Furthermore, renal dysfunction can affect the metabolism and clearance of antiplatelet and anticoagulant drugs, potentially causing drug accumulation and increased bleeding tendencies (29).

A severe reduction in LVEF results in a significant decrease in cardiac output and inadequate perfusion of systemic tissues, including the gastrointestinal tract. This condition can lead to mucosal ischemia, disruption of barrier function, and the formation of ulcers (30). Antiplatelet and anticoagulant therapies are necessary following AMI-PCI. However, a severe reduction in LVEF can prolong drug metabolism and increase cumulative exposure to these medications (31). Additionally, a significant reduction in LVEF is frequently linked to right heart failure. This can result in liver congestion, impaired liver function, and reduced production of coagulation factors—including II, VII, IX, and X—and platelets, thereby increasing the risk of bleeding (32). Research conducted by Jacob et al. (33) and others indicates that LVEF correlates with the SCAI-CS stage, with low LVEF being associated with a higher mortality rate.

The three indicators involved in this study are SCAI-CS stage, eGFR, and LVEF, which are easy to obtain and are suitable for all medical institutions.

### 4.3 Recent prognosis of patients with UGIB following emergency PCI for AMICS

Gastrointestinal bleeding after PCI can negatively impact the prognosis of patients with AMI and increase the risk of MACEs during the early stage, including hospitalization and within 30 days post-discharge. The in-hospital mortality rate among patients with gastrointestinal bleeding post-AMI is significantly higher than that of patients without gastrointestinal bleeding (14.3% vs. 2.1%, *P* = 0.047). Elevated hospital mortality is independently associated with peak TnI levels, the presence of CS, and the use of mechanical ventilators (20). Wang Lei et al. (34) also identified gastrointestinal bleeding as a risk factor for in-hospital mortality following emergency PCI in patients with AMICS.

This study is similar to the abolve study,demonstrating that the in-hospital mortality rate among patients with UGIB following emergency PCI for AMICS was significantly higher compared to patients without bleeding (63.8% vs. 29.7%, *P* < 0.05). Additionally, surviving patients in the bleeding group experienced significantly longer hospitalization durations than those in the non-bleeding group (*P* < 0.05). These findings suggest that UGIB after emergency PCI for AMICS is associated with poor prognosis, prolonged hospital stays, and increased financial burden. Therefore, preventing upper gastrointestinal bleeding in high-risk patients is of critical importance.

### 4.4 Prevention of UGIB patients following AMICS emergency PCI

Although the aforementioned risk factors are immutable, a comprehensive understanding of these factors enables standardized risk stratification and scientific management, facilitating the development of personalized antithrombotic plans. While the predictive model's specificity is only 52.3%, for a severe complication such as “AMICS emergency PCI postoperative UGIB” which has an in-hospital mortality rate of 63.8%, the primary goal of the model is to “maximize the identification of high-risk individuals to avoid missed diagnoses and prevent deaths,” rather than to pursue “absolute high specificity to reduce false diagnoses.” The core value of the model is “identifying high-risk individuals” and providing early prevention. PPIs elevate gastric juice pH by inhibiting gastric acid secretion, thereby promoting blood clot formation, stabilizing existing clots, and accelerating lesion healing (35). In the early stages, intravenous administration of PPIs, H_2_ receptor antagonists, and gastric mucosal protectors should be implemented to reduce the likelihood of bleeding (36).Given the high mortality rate among patients with UGIB, to enhance clinical translatability, we have developed and validated a simplified bedside scoring system. For example, assigning 1 point for each risk factor (SCAI-CS D + E = 1, LVEF < 36% = 1, eGFR < 45 = 1) creates a 0–3 point scale, with a score of ≥2 corresponding to high risk.Once high-risk patients have been identified, intravenous proton pump inhibitor (PPI) preparations should be administered promptly to prevent UGIB.

## 5 Study strengths

Firstly, by addressing a gap in existing research, we developed a composite indicator that integrates three complementary dimensions: the SCAI-CS stage (shock severity), LVEF (cardiac function), and eGFR (renal function).Unlike previous studies that relied on single-domain predictors, such as isolated eGFR or LVEF, our indicator captures the synergistic effects of multi-organ dysfunction on UGIB risk. This approach aligns with the clinical reality that AMICS-related UGIB is rarely driven by a single factor. For instance, our sensitivity analysis demonstrated that excluding any one of the three components reduced the model's AUC by 8.2%–11.5%, confirming the added value of this multi-dimensional integration.Secondly, the model exhibits strong clinical relevance and practicality. It utilizes only routine, bedside-measurable indicators: SCAI-CS stage is assessed via standard clinical examination, LVEF through rapid emergency echocardiography (a standard test for AMICS patients), and eGFR via point-of-care serum creatinine testing. Consequently, a single cardiologist can apply our model within 30 min of patient admission, addressing the “time-sensitive” need for UGIB risk stratification in AMICS patients undergoing emergency PCI. Additionally, we performed three different internal validations, including the Hosmer-Lemeshow test, the ROC curve, and the DCA of the predictive model, all of which indicated that the model offers good clinical utility.

## 6 Limitations

First, it is a single-center, retrospective cohort study, which may induce selection bias and information bias. Second, the relatively small patient cohort may reduce the statistical power, affecting the stability and reliability of the results, and limiting in-depth analysis of complex factor interactions. The third and most critical point is that this study did not conduct external validation of the predictive model, which is the primary factor limiting the general applicability of the model. Without external validation, we cannot confirm whether our model would perform similarly in non-third-level hospitals, regions with different racial compositions (such as areas with a higher prevalence of Helicobacter pylori infection), or populations with more comorbidities (such as patients with chronic liver diseases)—all of which may alter the risk of UGIB and the performance of the model. Due to practical operational limitations, we were unable to collect independent external sample groups. We are well aware that this is a significant limitation, which weakens the clinical generalizability of the model.

## 7 Future research recommendations

Firstly, the top priority is to validate the model in external, multi-center cohorts, including non-tertiary hospitals and regions with diverse ethnic and demographic characteristics. Secondly, to improve the model's moderate specificity (52.3%), future research should explore incorporating additional predictors, such as the use anticoagulants pre-PCI, genetic markers, and biomarkers of mucosal damage. A larger sample size (target n = 1,000) will enable multivariate analysis to determine which additional predictors most effectively improve specificity without reducing sensitivity.

## 8 Conclusions

This study constructed a predictive model, which demonstrates that high baseline SCAI-CS stage, poor kidney function, and low LVEF are independent risk factors for UGIB during hospitalization. The constructed predictive model demonstrates high predictive efficacy.

## Data Availability

The raw data supporting the conclusions of this article will be made available by the authors, without undue reservation.
